# {Dimeth­yl [(phenyl­sulfon­yl)amido­]phosphato-κ^2^ *O*,*O*′}bis­(tri­phenylphosphane-κ*P*)copper(I)

**DOI:** 10.1107/S1600536814010095

**Published:** 2014-05-17

**Authors:** Olesia V. Moroz, Viktor A. Trush, Tatiana Yu. Sliva, Kateryna O. Znovjyak, Vladimir M. Amirkhanov

**Affiliations:** a Taras Shevchenko National University of Kyiv, Department of Chemistry, 64/13 Volodymyrska Street, Kyiv 01601, Ukraine

## Abstract

In the title complex, [Cu(C_8_H_11_NO_5_PS)(C_18_H_15_P)_2_], the Cu^I^ ion is coordinated by two tri­phenyl­phosphane mol­ecules and two O atoms of the chelating dimeth­yl(phenyl­sulfon­yl)amido­phosphate anion, generating a squashed CuO_2_P_2_ tetrahedron. In the six-membered chelate ring, the Cu, P and O atoms are almost coplanar (r.m.s. deviation = 0.024 Å), with the N and S atoms displaced in the same direction, by 0.708 (5) and 0.429 (2) Å, respectively.

## Related literature   

For the synthesis of sulfonyl­amide derivatives, see: Kirsanov (1965 ▶); Moroz *et al.* (2012 ▶). For details of the pharmacological and biological properties of sulfonyl­amide derivatives, see: Kishino & Saito (1979 ▶); Xu & Angell (2000 ▶). For Cu^I^-containing complexes with tri­phenyl­phosphane, see: Barron *et al.* (1987 ▶); Yang *et al.* (2001 ▶); Zabirov *et al.* (2003 ▶). For details of potential applications of Cu^I^-containing complexes, see: Nagashima *et al.* (1993 ▶); Nondek *et al.* (1987 ▶); Tarkhanova *et al.* (2001 ▶); Zazybin *et al.* (2006 ▶); Verat *et al.* (2006 ▶). For coord­ination compounds of 3*d* metals with sulfonylamidophosphate ligands, see: Moroz *et al.* (2009 ▶); Trush *et al.* (2011 ▶). For the coordination mode of structural analogs of β-diketones, see: Gawryszewska *et al.* (2011 ▶); Yizhak *et al.* (2013 ▶); Kariaka *et al.* (2013 ▶); Amirkhanov *et al.* (2014 ▶).
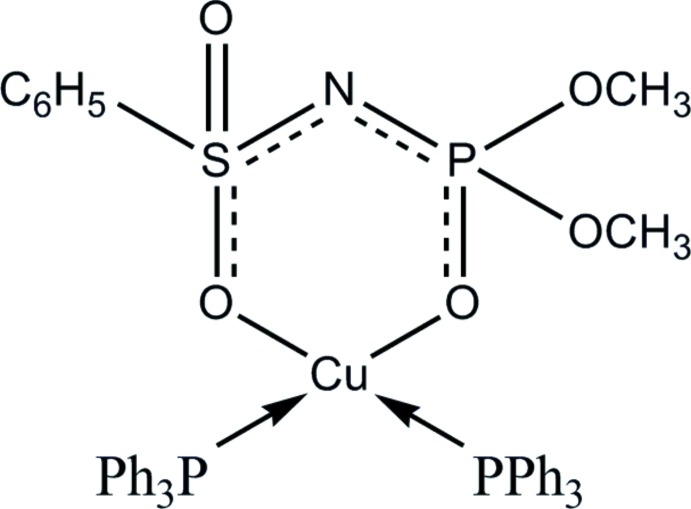


## Experimental   

### 

#### Crystal data  

[Cu(C_8_H_11_NO_5_PS)(C_18_H_15_P)_2_]
*M* *_r_* = 852.29
Monoclinic, 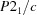

*a* = 12.8657 (12) Å
*b* = 26.281 (3) Å
*c* = 13.971 (3) Å
β = 121.875 (10)°
*V* = 4011.6 (11) Å^3^
*Z* = 4
Mo *K*α radiation
μ = 0.76 mm^−1^
*T* = 100 K
0.40 × 0.30 × 0.10 mm

#### Data collection  

Nonius KappaCCD diffractometer
Absorption correction: multi-scan (*SADABS*; Sheldrick, 2003 ▶) *T*_min_ = 0.74, *T*_max_ = 0.90
27134 measured reflections
9293 independent reflections
4739 reflections with *I* > 2σ(*I*)
*R*_int_ = 0.137

#### Refinement  

*R*[*F* ^2^ > 2σ(*F* ^2^)] = 0.086
*wR*(*F* ^2^) = 0.120
*S* = 1.04
9293 reflections
438 parameters
H-atom parameters constrained
Δρ_max_ = 0.48 e Å^−3^
Δρ_min_ = −0.56 e Å^−3^

### 

Data collection: *COLLECT* (Nonius, 1999 ▶); cell refinement: *DENZO*/*SCALEPACK* (Otwinowski & Minor, 1997 ▶); data reduction: *DENZO*/*SCALEPACK*; program(s) used to solve structure: *SHELXS97* (Sheldrick, 2008 ▶); program(s) used to refine structure: *SHELXL97* (Sheldrick, 2008 ▶); molecular graphics: *ORTEP-3 for Windows* (Farrugia, 2012 ▶); software used to prepare material for publication: *WinGX* (Farrugia, 2012 ▶).

## Supplementary Material

Crystal structure: contains datablock(s) I, cusp. DOI: 10.1107/S1600536814010095/hb7222sup1.cif

Structure factors: contains datablock(s) I. DOI: 10.1107/S1600536814010095/hb7222Isup2.hkl

CCDC reference: 1000731

Additional supporting information:  crystallographic information; 3D view; checkCIF report

## Figures and Tables

**Table 1:** Selected bond lengths (Å)

Cu1—O3	2.065 (3)
Cu1—O1	2.263 (3)
Cu1—P2	2.2345 (15)
Cu1—P3	2.2381 (14)

**Fig. 1.**
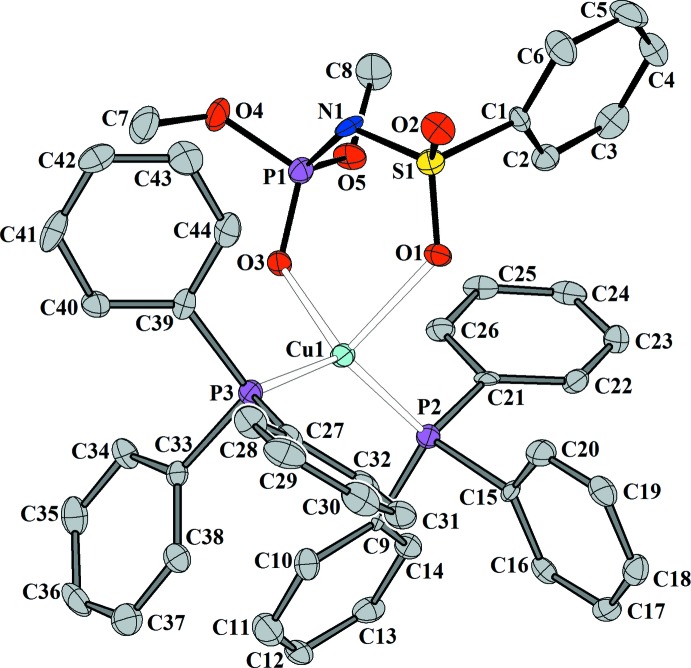
Structural representation of I with atom numbering scheme and 50% probability thermal ellipsoid. The H atoms are omitted for clarity.

## supplementary crystallographic information

### 1. Introduction

Coordination chemistry of structural analogs of *β*-diketones has been widely studied during last 30 years. Among them, sulfonyl phospho­ramides (SAPh) bearing a S(O)_2_NHP(O) structural fragment and different substituents at sulfur and phospho­rus atoms were first synthesized by Kirsanov (Kirsanov, 1965). These compounds are extensively used in medicine as bactericidal agents (Xu & Angell, 2000) and in agriculture as pesticides (Kishino & Saito, 1979). Recently, coordination compounds of lanthanides and 3*d* metals with SAPhs behaving as bidentate O,O-donor chelating ligands have been reported (Moroz, *et al.*, 2009; Moroz, *et al.*, 2012; Trush, *et al.*, 2011). As our contribution to the study of coordination compounds of 3*d* metals based on SAPh, we synthesized and structurally characterized a copper(I)-containing complex [Cu(*L*)(PPh_3_)_2_] (I) {where, H*L* is di­methyl­(phenyl­sulfonyl)­amido­phosphate (C_6_H_5_S(O)_2_NHP(O)(OCH_3_)_2_) and PPh_3_ is tri­phenyl­phosphane}. Complexes of Cu^I^ have useful luminescent properties, they can be used in microelectronics and as catalysts of homolytic C–Hal (Hal = Cl, Br) bond cleavage in polyhaloalkanes (Nagashima, *et al.*, 1993; Nondek, *et al.*, 1987; Tarkhanova, *et al.*, 2001; Zazybin, *et al.*, 2006). Tri­phenyl­phosphane molecule was used to prevent cluster formation during complexation reaction.

The molecular structure of I is shown in Figure1. Van der Waals contacts exist between molecules of I in the crystal structure. The coordination environment of the Cu ion is a distorted tetra­hedron (2+2). The values for the bond angles around the central atom are in the range from 88.9 (1)° to 129.94 (6)°. The coordination polyhedron consists of two phospho­rus atoms from PPh_3_ molecules and two oxygen atoms from the phospho­ryl and the sulfonyl groups of *L*^-^, which is coordinated in bidentate chelate mode forming with central ion a six-membered chelate ring. The later coordination mode is typical for the deprotonated structural analogs of *β*-diketones, SAPh and CAPh (carbacyl­amido­phosphates) (Gawryszewska, *et al.*, 2011; Yizhak, *et al.*, 2013; Kariaka, *et al.*, 2013; Amirkhanov, *et al.*, 2014). It has already been observed for [Cu(PPh_3_)_n_L_1_] based on N-acyl­amido­phosphinate ligands (Verat, *et al.*, 2006) and for (PPh_3_)_3_CuI (Barron, *et al.*, 1987) that the number of the coordinated PPh_3_ molecules to the central ion has the main influence on the Cu–P bond lengths. As in the present compound the Cu^I^ atom coordinates two PPh_3_, the Cu–P distances of 2.234 (1), 2.238 (1)Å are in good agreement with the values observed for the complexes containing two PPh_3_ ligands (Yang, *et al.*, 2001; Zabirov, *et al.*, 2003; Verat, *et al.*, 2006).

The Cu–O(S) and Cu–O(P) bond lengths (2.263 (3) and 2.066 (3)Å, respectively) (Table1) are similar to the reported values for the complexes of 3*d*-metals with H*L* (Moroz, *et al.*, 2009; Trush, *et al.*, 2011). The amide nitro­gen atom of the ligand is deprotonated that leads to decreasing the S–N, N–P and increasing the P–O, S–O bond length values (Table1) compared with those for H*L* (Moroz, *et al.*, 2009). Such changes may be related to the occurrence of the π-coupling in O—S—N—P—O fragment and are usual for SAPh ligands (Moroz, *et al.*, 2009; Trush, *et al.*, 2011). The value of the O(1)—Cu(1)—O(3) angle of 88.9 (1)° is typical for the coordination compounds with O- donor SAPh and CAPh ligands. The phospho­rus and sulfur atoms of I have distorted tetra­hedral configurations (Table1). Oxygen atoms of the phospho­ryl O(3) and the sulfonyl O(2) groups of the O—S—N—P—O structural fragment adopt an anti­clinal conformation (the dihedral angle between O(2)—S(1)—P(1) and O(3)—P(1)—S(1) planes is 106.3°). The six-membered Cu—O—P—N—S—O metallocycle has a distorted boat conformation with the deviations of N(1) and Cu(1) from the mean plane of 0.40Å and 0.26Å, respectively.

### 2. Experimental

The synthesis of H*L* was carried out according to previously published procedure (Moroz, *et al.*, 2009).

Compound I was prepared according to the following scheme:

4H*L* + 4Cu + 8PPh_3_ + O_2_→ 4[Cu(*L*)(PPh_3_)_2_] + 2H_2_O

Briefly, a heterogeneous mixture of copper powder (0.065g, 1mmol), tri­phenyl­phosphane (0.524g, 2mmol) and H*L* (0.287g, 1mmol) in 40mL of acetone was stirred for 4 days upon heating with a reflux condenser. Decreasing of the powder was observed simultaneously with gradual appearance of white precipitate. The later was filtered off, washed thoroughly with chilled iso­propanol, dried and dissolved in minimal amount of DMF to remove the residual copper powder. The resulting solution was left at ambient temperature for crystallization in air. The colorless crystals were collected by filtration after 2 days, washed with chilled iso­propanol and dried on filter. Yield: 0.64g (75%). The compound is soluble in methanol, aceto­nitrile, chloro­form, DMSO and DMF. Anal. calc. for C_44_H_41_NO_5_P_3_SCu: C61.81, H4.71, N1.61, S3.54%; found: C62.00, H4.85, N1.64, S3.76%; IR (KBr, cm^-1^): 1225, 1250 (s, SO_2_) and 1180 (s, PO); ^1^H NMR (400 MHz, CDCl_3_): 3.28 (d, 6H, CH_3_, ^3^*J*_P–H_ = 12Hz), 7.09 (m, H_β_, 2H, C_6_H_5_(*L*^-^)), 7.27 (m, H_β_, 12H, C_6_H_5_(PPh_3_)), 7.33 (s, H_γ_, 1H, C_6_H_5_(*L*^-^)), 7.39 (m, H_α__+__γ_, 18H, C_6_H_5_(PPh_3_)) 7.62 (m, H_α_, 2H, C_6_H_5_(*L*^-^)) ppm. ^31^P NMR (162.1 MHz, CDCl_3_): 0.8 (g, ^3^*J*_P–H_ = 12Hz, *L*^-^), -4.3 (s, PPh_3_) ppm.

### 3. Refinement

The H atoms were attached to carbon atoms geometrically. The H atoms were refined with riding constraints (C—H in the range 0.93–0.98Å, and U_iso_(H) lie in the range 1.2-1.5 times U_eq_ of the parent atom). Crystal data, data collection and structure refinement details are summarized in Table 1.

### Crystal data

**Table:**

[Cu(C_8_H_11_NO_5_PS)(C_18_H_15_P)_2_]	*F*(000) = 1768
*M**_r_* = 852.29	*D*_x_ = 1.411 Mg m^−^^3^
Monoclinic, *P*2_1_/*c*	Mo *K*α radiation, λ = 0.71073 Å
*a* = 12.8657 (12) Å	Cell parameters from 27134 reflections
*b* = 26.281 (3) Å	θ = 2.9–28.5°
*c* = 13.971 (3) Å	µ = 0.76 mm^−^^1^
β = 121.875 (10)°	*T* = 100 K
*V* = 4011.6 (11) Å^3^	Needle, colourless
*Z* = 4	0.40 × 0.30 × 0.10 mm

### Data collection

**Table:**

Nonius KappaCCD diffractometer	9293 independent reflections
Radiation source: sealed X-ray tube	4739 reflections with *I* > 2σ(*I*)
Detector resolution: 9 pixels mm^-1^	*R*_int_ = 0.137
φ scans and ω scans with κ offset	θ_max_ = 28.5°, θ_min_ = 2.9°
Absorption correction: multi-scan (*SADABS*; Sheldrick, 2003)	*h* = −16→16
*T*_min_ = 0.74, *T*_max_ = 0.90	*k* = −33→34
27134 measured reflections	*l* = −18→18

### Refinement

**Table:**

Refinement on *F*^2^	0 restraints
Least-squares matrix: full	Hydrogen site location: inferred from neighbouring sites
*R*[*F*^2^ > 2σ(*F*^2^)] = 0.086	H-atom parameters constrained
*wR*(*F*^2^) = 0.120	*w* = 1/[σ^2^(*F*_o_^2^) + (0.0188*P*)^2^] where *P* = (*F*_o_^2^ + 2*F*_c_^2^)/3
*S* = 1.04	(Δ/σ)_max_ = 0.001
9293 reflections	Δρ_max_ = 0.48 e Å^−^^3^
438 parameters	Δρ_min_ = −0.56 e Å^−^^3^

### Special details

**Table:**

Geometry. All e.s.d.'s (except the e.s.d. in the dihedral angle between two l.s. planes) are estimated using the full covariance matrix. The cell e.s.d.'s are taken into account individually in the estimation of e.s.d.'s in distances, angles and torsion angles; correlations between e.s.d.'s in cell parameters are only used when they are defined by crystal symmetry. An approximate (isotropic) treatment of cell e.s.d.'s is used for estimating e.s.d.'s involving l.s. planes.
Refinement. Refinement of *F*^2^ against ALL reflections. The weighted *R*-factor *wR* and goodness of fit *S* are based on *F*^2^, conventional *R*-factors *R* are based on *F*, with *F* set to zero for negative *F*^2^. The threshold expression of *F*^2^ > 2σ(*F*^2^) is used only for calculating *R*-factors(gt) *etc*. and is not relevant to the choice of reflections for refinement. *R*-factors based on *F*^2^ are statistically about twice as large as those based on *F*, and *R*- factors based on ALL data will be even larger.

### Fractional atomic coordinates and isotropic or equivalent isotropic displacement parameters (Å^2^)

**Table:**

	*x*	*y*	*z*	*U*_iso_*/*U*_eq_	
C1	0.7620 (4)	0.16461 (19)	1.0173 (4)	0.0134 (12)	
C2	0.7765 (5)	0.15558 (19)	0.9275 (4)	0.0195 (13)	
H68	0.7094	0.1479	0.8566	0.023*	
C3	0.8930 (5)	0.1582 (2)	0.9452 (5)	0.0257 (14)	
H102	0.9034	0.1531	0.8848	0.031*	
C4	0.9933 (5)	0.1682 (2)	1.0499 (5)	0.0260 (14)	
H106	1.0707	0.1698	1.0602	0.031*	
C5	0.9789 (5)	0.1758 (2)	1.1401 (5)	0.0323 (16)	
H19	1.0468	0.1822	1.2115	0.039*	
C6	0.8614 (5)	0.1740 (2)	1.1237 (5)	0.0277 (15)	
H57	0.8509	0.1790	1.1840	0.033*	
C7	0.3285 (5)	0.2987 (2)	0.8180 (5)	0.0375 (17)	
H74A	0.2652	0.2749	0.7708	0.056*	
H74B	0.3002	0.3327	0.7928	0.056*	
H74C	0.3493	0.2948	0.8945	0.056*	
C8	0.6702 (5)	0.2749 (2)	0.8269 (5)	0.0303 (15)	
H7A	0.6423	0.3079	0.8330	0.045*	
H7B	0.7084	0.2774	0.7838	0.045*	
H7C	0.7283	0.2622	0.9008	0.045*	
C9	0.2961 (5)	0.06859 (19)	0.5000 (4)	0.0203 (5)	
C10	0.1706 (5)	0.06746 (19)	0.4622 (4)	0.0203 (5)	
H25	0.1469	0.0701	0.5144	0.024*	
C11	0.0819 (5)	0.06245 (18)	0.3490 (4)	0.0203 (5)	
H21	−0.0003	0.0612	0.3260	0.024*	
C12	0.1160 (5)	0.05931 (19)	0.2692 (4)	0.0203 (5)	
H16	0.0567	0.0560	0.1930	0.024*	
C13	0.2384 (5)	0.06113 (19)	0.3045 (4)	0.0203 (5)	
H15	0.2613	0.0599	0.2515	0.024*	
C14	0.3274 (5)	0.06484 (19)	0.4179 (4)	0.0203 (5)	
H35	0.4095	0.0648	0.4403	0.024*	
C15	0.4477 (4)	0.00831 (18)	0.6988 (4)	0.0132 (12)	
C16	0.4271 (4)	−0.03269 (19)	0.6270 (4)	0.0147 (12)	
H20	0.3927	−0.0268	0.5504	0.018*	
C17	0.4570 (5)	−0.0819 (2)	0.6678 (5)	0.0189 (13)	
H6	0.4437	−0.1088	0.6192	0.023*	
C18	0.5070 (5)	−0.0910 (2)	0.7817 (5)	0.0215 (14)	
H56	0.5264	−0.1241	0.8093	0.026*	
C19	0.5283 (5)	−0.0510 (2)	0.8547 (5)	0.0242 (14)	
H8	0.5628	−0.0573	0.9311	0.029*	
C20	0.4987 (5)	−0.0024 (2)	0.8145 (5)	0.0236 (14)	
H31	0.5123	0.0241	0.8640	0.028*	
C21	0.5434 (5)	0.09651 (19)	0.6489 (4)	0.0141 (12)	
C22	0.6453 (5)	0.0664 (2)	0.6779 (4)	0.0184 (13)	
H60	0.6493	0.0333	0.7032	0.022*	
C23	0.7411 (5)	0.0854 (2)	0.6694 (4)	0.0229 (14)	
H29	0.8085	0.0650	0.6888	0.027*	
C24	0.7358 (5)	0.1347 (2)	0.6320 (4)	0.0243 (14)	
H54	0.7988	0.1472	0.6245	0.029*	
C25	0.6370 (5)	0.1655 (2)	0.6057 (4)	0.0191 (13)	
H70	0.6346	0.1988	0.5822	0.023*	
C26	0.5425 (5)	0.14690 (19)	0.6142 (4)	0.0191 (13)	
H67	0.4767	0.1680	0.5966	0.023*	
C27	0.1898 (4)	0.0599 (2)	0.8496 (4)	0.0177 (13)	
C28	0.1400 (5)	0.0630 (2)	0.9169 (4)	0.0232 (14)	
H105	0.1078	0.0938	0.9224	0.028*	
C29	0.1375 (5)	0.0207 (2)	0.9761 (5)	0.0324 (16)	
H1	0.1021	0.0229	1.0193	0.039*	
C30	0.1891 (5)	−0.0249 (2)	0.9696 (5)	0.0332 (16)	
H45	0.1901	−0.0531	1.0104	0.040*	
C31	0.2386 (5)	−0.0284 (2)	0.9029 (5)	0.0285 (15)	
H72	0.2710	−0.0591	0.8975	0.034*	
C32	0.2400 (5)	0.01393 (19)	0.8440 (4)	0.0209 (14)	
H38	0.2748	0.0115	0.8003	0.025*	
C33	0.0584 (4)	0.11361 (19)	0.6394 (4)	0.0143 (12)	
C34	0.0297 (5)	0.1559 (2)	0.5668 (4)	0.0191 (13)	
H61	0.0792	0.1847	0.5923	0.023*	
C35	−0.0716 (5)	0.1547 (2)	0.4583 (5)	0.0251 (15)	
H107	−0.0912	0.1832	0.4124	0.030*	
C36	−0.1443 (5)	0.1115 (2)	0.4168 (5)	0.0275 (15)	
H47	−0.2106	0.1104	0.3429	0.033*	
C37	−0.1168 (5)	0.0703 (2)	0.4865 (5)	0.0275 (15)	
H66	−0.1659	0.0415	0.4594	0.033*	
C38	−0.0173 (5)	0.0709 (2)	0.5966 (4)	0.0194 (13)	
H62	−0.0008	0.0425	0.6423	0.023*	
C39	0.1906 (5)	0.1685 (2)	0.8525 (5)	0.0235 (6)	
C40	0.0861 (5)	0.19774 (19)	0.8175 (5)	0.0235 (6)	
H13	0.0132	0.1899	0.7510	0.028*	
C41	0.0923 (5)	0.2386 (2)	0.8830 (4)	0.0235 (6)	
H28	0.0230	0.2585	0.8589	0.028*	
C42	0.1968 (5)	0.2504 (2)	0.9812 (5)	0.0235 (6)	
H65	0.1984	0.2777	1.0242	0.028*	
C43	0.3019 (5)	0.2214 (2)	1.0173 (5)	0.0235 (6)	
H14	0.3742	0.2291	1.0845	0.028*	
C44	0.2972 (5)	0.1810 (2)	0.9521 (4)	0.0235 (6)	
H75	0.3673	0.1617	0.9757	0.028*	
Cu1	0.36062 (6)	0.12135 (2)	0.75302 (5)	0.01638 (18)	
N1	0.5554 (4)	0.21783 (15)	0.9479 (3)	0.0169 (10)	
O1	0.5448 (3)	0.12269 (13)	0.9166 (3)	0.0198 (9)	
O2	0.6296 (3)	0.15752 (13)	1.1063 (3)	0.0246 (9)	
O3	0.3780 (3)	0.19846 (12)	0.7364 (3)	0.0173 (9)	
O4	0.4335 (3)	0.28906 (12)	0.8119 (3)	0.0268 (10)	
O5	0.5671 (3)	0.24042 (13)	0.7711 (3)	0.0227 (9)	
P1	0.48025 (13)	0.23250 (5)	0.81736 (13)	0.0190 (4)	
P2	0.41040 (12)	0.07398 (5)	0.65026 (12)	0.0153 (3)	
P3	0.20168 (12)	0.11467 (5)	0.77600 (12)	0.0162 (3)	
S1	0.61185 (12)	0.16414 (5)	0.99649 (12)	0.0183 (3)	

### Atomic displacement parameters (Å^2^)

**Table:**

	*U*^11^	*U*^22^	*U*^33^	*U*^12^	*U*^13^	*U*^23^
C1	0.008 (3)	0.014 (3)	0.015 (3)	0.000 (2)	0.004 (3)	0.003 (2)
C2	0.022 (4)	0.019 (3)	0.018 (3)	−0.001 (3)	0.011 (3)	−0.001 (3)
C3	0.034 (4)	0.026 (3)	0.026 (4)	0.003 (3)	0.022 (3)	0.001 (3)
C4	0.015 (3)	0.032 (4)	0.027 (4)	0.002 (3)	0.008 (3)	0.001 (3)
C5	0.021 (4)	0.045 (4)	0.023 (4)	−0.007 (3)	0.006 (3)	−0.013 (3)
C6	0.022 (4)	0.042 (4)	0.021 (4)	0.000 (3)	0.013 (3)	−0.002 (3)
C7	0.029 (4)	0.025 (4)	0.067 (5)	0.002 (3)	0.031 (4)	−0.005 (3)
C8	0.025 (4)	0.027 (4)	0.041 (4)	−0.007 (3)	0.020 (3)	0.008 (3)
C9	0.0184 (14)	0.0199 (12)	0.0188 (14)	0.0008 (11)	0.0072 (12)	−0.0007 (11)
C10	0.0184 (14)	0.0199 (12)	0.0188 (14)	0.0008 (11)	0.0072 (12)	−0.0007 (11)
C11	0.0184 (14)	0.0199 (12)	0.0188 (14)	0.0008 (11)	0.0072 (12)	−0.0007 (11)
C12	0.0184 (14)	0.0199 (12)	0.0188 (14)	0.0008 (11)	0.0072 (12)	−0.0007 (11)
C13	0.0184 (14)	0.0199 (12)	0.0188 (14)	0.0008 (11)	0.0072 (12)	−0.0007 (11)
C14	0.0184 (14)	0.0199 (12)	0.0188 (14)	0.0008 (11)	0.0072 (12)	−0.0007 (11)
C15	0.011 (3)	0.010 (3)	0.022 (3)	0.000 (2)	0.011 (3)	−0.004 (2)
C16	0.012 (3)	0.020 (3)	0.014 (3)	−0.002 (2)	0.008 (3)	0.002 (3)
C17	0.018 (3)	0.018 (3)	0.027 (4)	−0.005 (3)	0.016 (3)	−0.006 (3)
C18	0.020 (3)	0.019 (3)	0.028 (4)	0.004 (3)	0.015 (3)	0.002 (3)
C19	0.019 (3)	0.029 (4)	0.013 (3)	0.002 (3)	0.000 (3)	0.002 (3)
C20	0.025 (4)	0.021 (3)	0.017 (3)	0.002 (3)	0.005 (3)	−0.003 (3)
C21	0.012 (3)	0.018 (3)	0.010 (3)	−0.004 (2)	0.005 (3)	−0.008 (2)
C22	0.019 (3)	0.015 (3)	0.026 (4)	−0.001 (3)	0.016 (3)	−0.003 (3)
C23	0.016 (3)	0.024 (3)	0.028 (4)	0.001 (3)	0.011 (3)	−0.006 (3)
C24	0.027 (4)	0.030 (4)	0.022 (3)	−0.013 (3)	0.017 (3)	−0.007 (3)
C25	0.023 (4)	0.021 (3)	0.018 (3)	−0.010 (3)	0.015 (3)	−0.005 (3)
C26	0.020 (3)	0.015 (3)	0.021 (3)	−0.004 (3)	0.010 (3)	−0.003 (3)
C27	0.011 (3)	0.021 (3)	0.017 (3)	−0.004 (3)	0.005 (3)	0.000 (3)
C28	0.020 (3)	0.024 (3)	0.025 (4)	−0.002 (3)	0.011 (3)	0.006 (3)
C29	0.028 (4)	0.037 (4)	0.031 (4)	−0.014 (3)	0.014 (3)	−0.001 (3)
C30	0.033 (4)	0.024 (4)	0.025 (4)	−0.007 (3)	0.004 (3)	0.015 (3)
C31	0.023 (4)	0.021 (4)	0.033 (4)	−0.003 (3)	0.009 (3)	0.000 (3)
C32	0.013 (3)	0.017 (3)	0.017 (3)	−0.001 (3)	−0.002 (3)	0.004 (3)
C33	0.013 (3)	0.015 (3)	0.018 (3)	0.000 (2)	0.011 (3)	−0.002 (3)
C34	0.010 (3)	0.027 (3)	0.025 (4)	−0.003 (3)	0.012 (3)	0.002 (3)
C35	0.019 (4)	0.032 (4)	0.029 (4)	0.006 (3)	0.015 (3)	0.007 (3)
C36	0.011 (3)	0.045 (4)	0.023 (4)	−0.002 (3)	0.007 (3)	−0.003 (3)
C37	0.023 (4)	0.026 (4)	0.035 (4)	−0.002 (3)	0.016 (3)	−0.006 (3)
C38	0.013 (3)	0.024 (3)	0.019 (3)	0.000 (3)	0.008 (3)	0.000 (3)
C39	0.0276 (15)	0.0243 (14)	0.0243 (15)	0.0026 (12)	0.0176 (13)	−0.0019 (11)
C40	0.0276 (15)	0.0243 (14)	0.0243 (15)	0.0026 (12)	0.0176 (13)	−0.0019 (11)
C41	0.0276 (15)	0.0243 (14)	0.0243 (15)	0.0026 (12)	0.0176 (13)	−0.0019 (11)
C42	0.0276 (15)	0.0243 (14)	0.0243 (15)	0.0026 (12)	0.0176 (13)	−0.0019 (11)
C43	0.0276 (15)	0.0243 (14)	0.0243 (15)	0.0026 (12)	0.0176 (13)	−0.0019 (11)
C44	0.0276 (15)	0.0243 (14)	0.0243 (15)	0.0026 (12)	0.0176 (13)	−0.0019 (11)
Cu1	0.0157 (4)	0.0148 (4)	0.0206 (4)	−0.0015 (3)	0.0109 (3)	−0.0025 (3)
N1	0.025 (3)	0.009 (2)	0.017 (3)	−0.002 (2)	0.012 (2)	−0.004 (2)
O1	0.016 (2)	0.016 (2)	0.021 (2)	−0.0027 (18)	0.0045 (18)	−0.0015 (18)
O2	0.027 (2)	0.030 (2)	0.019 (2)	−0.0022 (19)	0.014 (2)	0.0023 (19)
O3	0.014 (2)	0.012 (2)	0.024 (2)	−0.0024 (17)	0.0090 (19)	0.0023 (17)
O4	0.024 (2)	0.012 (2)	0.041 (3)	0.0049 (18)	0.016 (2)	0.0027 (19)
O5	0.021 (2)	0.022 (2)	0.028 (2)	−0.0081 (18)	0.015 (2)	−0.0018 (19)
P1	0.0175 (9)	0.0135 (8)	0.0252 (9)	−0.0002 (7)	0.0108 (8)	0.0006 (7)
P2	0.0143 (8)	0.0137 (8)	0.0186 (9)	−0.0004 (6)	0.0093 (7)	−0.0005 (7)
P3	0.0144 (8)	0.0150 (8)	0.0211 (8)	−0.0007 (7)	0.0106 (7)	−0.0002 (7)
S1	0.0198 (9)	0.0175 (8)	0.0189 (8)	−0.0015 (7)	0.0110 (7)	0.0002 (7)

### Geometric parameters (Å, º)

**Table:**

C1—C6	1.378 (7)	C24—H54	0.9300
C1—C2	1.384 (6)	C25—C26	1.373 (6)
C1—S1	1.795 (5)	C25—H70	0.9300
C2—C3	1.386 (7)	C26—H67	0.9300
C2—H68	0.9300	C27—C28	1.392 (6)
C3—C4	1.371 (7)	C27—C32	1.392 (7)
C3—H102	0.9300	C27—P3	1.821 (5)
C4—C5	1.381 (7)	C28—C29	1.396 (7)
C4—H106	0.9300	C28—H105	0.9300
C5—C6	1.405 (7)	C29—C30	1.396 (7)
C5—H19	0.9300	C29—H1	0.9300
C6—H57	0.9300	C30—C31	1.382 (7)
C7—O4	1.419 (5)	C30—H45	0.9300
C7—H74A	0.9600	C31—C32	1.390 (7)
C7—H74B	0.9600	C31—H72	0.9300
C7—H74C	0.9600	C32—H38	0.9300
C8—O5	1.448 (5)	C33—C38	1.397 (6)
C8—H7A	0.9600	C33—C34	1.416 (6)
C8—H7B	0.9600	C33—P3	1.824 (5)
C8—H7C	0.9600	C34—C35	1.382 (7)
C9—C14	1.406 (6)	C34—H61	0.9300
C9—C10	1.409 (6)	C35—C36	1.390 (7)
C9—P2	1.824 (5)	C35—H107	0.9300
C10—C11	1.383 (6)	C36—C37	1.370 (7)
C10—H25	0.9300	C36—H47	0.9300
C11—C12	1.400 (6)	C37—C38	1.387 (7)
C11—H21	0.9300	C37—H66	0.9300
C12—C13	1.380 (6)	C38—H62	0.9300
C12—H16	0.9300	C39—C44	1.382 (7)
C13—C14	1.384 (6)	C39—C40	1.394 (7)
C13—H15	0.9300	C39—P3	1.823 (5)
C14—H35	0.9300	C40—C41	1.386 (7)
C15—C16	1.399 (6)	C40—H13	0.9300
C15—C20	1.415 (7)	C41—C42	1.355 (7)
C15—P2	1.824 (5)	C41—H28	0.9300
C16—C17	1.385 (6)	C42—C43	1.394 (6)
C16—H20	0.9300	C42—H65	0.9300
C17—C18	1.386 (7)	C43—C44	1.380 (7)
C17—H6	0.9300	C43—H14	0.9300
C18—C19	1.388 (7)	C44—H75	0.9300
C18—H56	0.9300	Cu1—O3	2.065 (3)
C19—C20	1.366 (7)	Cu1—O1	2.263 (3)
C19—H8	0.9300	Cu1—P2	2.2345 (15)
C20—H31	0.9300	Cu1—P3	2.2381 (14)
C21—C22	1.395 (6)	N1—S1	1.568 (4)
C21—C26	1.409 (6)	N1—P1	1.596 (4)
C21—P2	1.820 (5)	O1—S1	1.470 (3)
C22—C23	1.391 (6)	O2—S1	1.438 (3)
C22—H60	0.9300	O3—P1	1.496 (3)
C23—C24	1.386 (7)	O4—P1	1.590 (3)
C23—H29	0.9300	O5—P1	1.572 (3)
C24—C25	1.382 (7)		
			
C6—C1—C2	121.0 (5)	C27—C28—C29	121.1 (5)
C6—C1—S1	118.9 (4)	C27—C28—H105	119.5
C2—C1—S1	120.1 (4)	C29—C28—H105	119.5
C1—C2—C3	118.8 (5)	C28—C29—C30	119.0 (5)
C1—C2—H68	120.6	C28—C29—H1	120.5
C3—C2—H68	120.6	C30—C29—H1	120.5
C4—C3—C2	121.2 (5)	C31—C30—C29	120.3 (5)
C4—C3—H102	119.4	C31—C30—H45	119.8
C2—C3—H102	119.4	C29—C30—H45	119.8
C3—C4—C5	119.9 (5)	C30—C31—C32	120.1 (5)
C3—C4—H106	120.1	C30—C31—H72	120.0
C5—C4—H106	120.1	C32—C31—H72	120.0
C4—C5—C6	119.8 (6)	C31—C32—C27	120.7 (5)
C4—C5—H19	120.1	C31—C32—H38	119.7
C6—C5—H19	120.1	C27—C32—H38	119.7
C1—C6—C5	119.2 (5)	C38—C33—C34	117.7 (5)
C1—C6—H57	120.4	C38—C33—P3	123.5 (4)
C5—C6—H57	120.4	C34—C33—P3	118.3 (4)
O4—C7—H74A	109.5	C35—C34—C33	120.4 (5)
O4—C7—H74B	109.5	C35—C34—H61	119.8
H74A—C7—H74B	109.5	C33—C34—H61	119.8
O4—C7—H74C	109.5	C34—C35—C36	120.8 (5)
H74A—C7—H74C	109.5	C34—C35—H107	119.6
H74B—C7—H74C	109.5	C36—C35—H107	119.6
O5—C8—H7A	109.5	C37—C36—C35	119.0 (5)
O5—C8—H7B	109.5	C37—C36—H47	120.5
H7A—C8—H7B	109.5	C35—C36—H47	120.5
O5—C8—H7C	109.5	C36—C37—C38	121.3 (5)
H7A—C8—H7C	109.5	C36—C37—H66	119.3
H7B—C8—H7C	109.5	C38—C37—H66	119.3
C14—C9—C10	117.3 (5)	C37—C38—C33	120.6 (5)
C14—C9—P2	122.7 (4)	C37—C38—H62	119.7
C10—C9—P2	120.1 (4)	C33—C38—H62	119.7
C11—C10—C9	121.3 (5)	C44—C39—C40	118.9 (5)
C11—C10—H25	119.3	C44—C39—P3	115.5 (4)
C9—C10—H25	119.3	C40—C39—P3	125.6 (4)
C10—C11—C12	120.0 (5)	C41—C40—C39	119.2 (5)
C10—C11—H21	120.0	C41—C40—H13	120.4
C12—C11—H21	120.0	C39—C40—H13	120.4
C13—C12—C11	119.5 (5)	C42—C41—C40	121.7 (5)
C13—C12—H16	120.3	C42—C41—H28	119.1
C11—C12—H16	120.3	C40—C41—H28	119.1
C12—C13—C14	120.6 (5)	C41—C42—C43	119.7 (5)
C12—C13—H15	119.7	C41—C42—H65	120.1
C14—C13—H15	119.7	C43—C42—H65	120.1
C13—C14—C9	121.3 (5)	C44—C43—C42	119.0 (5)
C13—C14—H35	119.4	C44—C43—H14	120.5
C9—C14—H35	119.4	C42—C43—H14	120.5
C16—C15—C20	117.6 (5)	C43—C44—C39	121.5 (5)
C16—C15—P2	123.3 (4)	C43—C44—H75	119.3
C20—C15—P2	119.0 (4)	C39—C44—H75	119.3
C17—C16—C15	121.2 (5)	O3—Cu1—P2	112.93 (9)
C17—C16—H20	119.4	O3—Cu1—P3	104.75 (9)
C15—C16—H20	119.4	P2—Cu1—P3	129.95 (6)
C16—C17—C18	119.6 (5)	O3—Cu1—O1	88.88 (13)
C16—C17—H6	120.2	P2—Cu1—O1	98.93 (9)
C18—C17—H6	120.2	P3—Cu1—O1	113.89 (9)
C17—C18—C19	120.3 (5)	S1—N1—P1	125.1 (3)
C17—C18—H56	119.9	S1—O1—Cu1	131.3 (2)
C19—C18—H56	119.9	P1—O3—Cu1	127.3 (2)
C20—C19—C18	120.2 (5)	C7—O4—P1	120.7 (3)
C20—C19—H8	119.9	C8—O5—P1	120.7 (3)
C18—C19—H8	119.9	O3—P1—O5	107.5 (2)
C19—C20—C15	121.1 (5)	O3—P1—O4	111.3 (2)
C19—C20—H31	119.5	O5—P1—O4	100.9 (2)
C15—C20—H31	119.5	O3—P1—N1	118.7 (2)
C22—C21—C26	117.9 (5)	O5—P1—N1	111.6 (2)
C22—C21—P2	123.9 (4)	O4—P1—N1	105.4 (2)
C26—C21—P2	118.2 (4)	C21—P2—C15	104.3 (2)
C23—C22—C21	120.7 (5)	C21—P2—C9	101.8 (2)
C23—C22—H60	119.6	C15—P2—C9	104.4 (2)
C21—C22—H60	119.6	C21—P2—Cu1	114.54 (16)
C24—C23—C22	119.9 (5)	C15—P2—Cu1	113.38 (17)
C24—C23—H29	120.1	C9—P2—Cu1	116.87 (17)
C22—C23—H29	120.1	C27—P3—C39	103.1 (2)
C25—C24—C23	120.2 (5)	C27—P3—C33	103.8 (2)
C25—C24—H54	119.9	C39—P3—C33	106.1 (2)
C23—C24—H54	119.9	C27—P3—Cu1	119.89 (17)
C26—C25—C24	120.1 (5)	C39—P3—Cu1	112.55 (18)
C26—C25—H70	120.0	C33—P3—Cu1	110.26 (16)
C24—C25—H70	120.0	O2—S1—O1	114.8 (2)
C25—C26—C21	121.2 (5)	O2—S1—N1	110.3 (2)
C25—C26—H67	119.4	O1—S1—N1	112.7 (2)
C21—C26—H67	119.4	O2—S1—C1	106.1 (2)
C28—C27—C32	118.8 (5)	O1—S1—C1	106.2 (2)
C28—C27—P3	122.7 (4)	N1—S1—C1	106.1 (2)
C32—C27—P3	118.3 (4)		
			
C6—C1—C2—C3	−2.7 (8)	Cu1—O3—P1—N1	−27.4 (3)
S1—C1—C2—C3	177.4 (4)	C8—O5—P1—O3	175.3 (3)
C1—C2—C3—C4	1.8 (8)	C8—O5—P1—O4	58.6 (4)
C2—C3—C4—C5	0.0 (8)	C8—O5—P1—N1	−53.0 (4)
C3—C4—C5—C6	−0.8 (9)	C7—O4—P1—O3	49.6 (4)
C2—C1—C6—C5	1.9 (8)	C7—O4—P1—O5	163.5 (4)
S1—C1—C6—C5	−178.2 (4)	C7—O4—P1—N1	−80.3 (4)
C4—C5—C6—C1	−0.1 (8)	S1—N1—P1—O3	49.7 (4)
C14—C9—C10—C11	0.5 (7)	S1—N1—P1—O5	−76.2 (3)
P2—C9—C10—C11	−178.8 (4)	S1—N1—P1—O4	175.1 (3)
C9—C10—C11—C12	−1.2 (8)	C22—C21—P2—C15	1.3 (5)
C10—C11—C12—C13	0.1 (8)	C26—C21—P2—C15	179.7 (4)
C11—C12—C13—C14	1.5 (8)	C22—C21—P2—C9	−107.1 (4)
C12—C13—C14—C9	−2.2 (8)	C26—C21—P2—C9	71.4 (4)
C10—C9—C14—C13	1.2 (8)	C22—C21—P2—Cu1	125.8 (4)
P2—C9—C14—C13	−179.6 (4)	C26—C21—P2—Cu1	−55.7 (4)
C20—C15—C16—C17	−0.8 (7)	C16—C15—P2—C21	−85.1 (4)
P2—C15—C16—C17	180.0 (4)	C20—C15—P2—C21	95.7 (4)
C15—C16—C17—C18	0.7 (7)	C16—C15—P2—C9	21.4 (5)
C16—C17—C18—C19	−0.7 (7)	C20—C15—P2—C9	−157.8 (4)
C17—C18—C19—C20	0.8 (8)	C16—C15—P2—Cu1	149.7 (4)
C18—C19—C20—C15	−0.9 (8)	C20—C15—P2—Cu1	−29.5 (4)
C16—C15—C20—C19	0.9 (8)	C14—C9—P2—C21	20.4 (5)
P2—C15—C20—C19	−179.9 (4)	C10—C9—P2—C21	−160.3 (4)
C26—C21—C22—C23	−1.8 (7)	C14—C9—P2—C15	−87.9 (5)
P2—C21—C22—C23	176.6 (4)	C10—C9—P2—C15	91.3 (4)
C21—C22—C23—C24	0.1 (8)	C14—C9—P2—Cu1	146.0 (4)
C22—C23—C24—C25	1.5 (8)	C10—C9—P2—Cu1	−34.8 (5)
C23—C24—C25—C26	−1.4 (8)	C28—C27—P3—C39	20.0 (5)
C24—C25—C26—C21	−0.3 (8)	C32—C27—P3—C39	−155.7 (4)
C22—C21—C26—C25	1.9 (7)	C28—C27—P3—C33	−90.5 (5)
P2—C21—C26—C25	−176.6 (4)	C32—C27—P3—C33	93.8 (4)
C32—C27—C28—C29	−1.4 (8)	C28—C27—P3—Cu1	146.0 (4)
P3—C27—C28—C29	−177.0 (4)	C32—C27—P3—Cu1	−29.7 (5)
C27—C28—C29—C30	1.7 (8)	C44—C39—P3—C27	80.6 (4)
C28—C29—C30—C31	−1.8 (8)	C40—C39—P3—C27	−100.9 (5)
C29—C30—C31—C32	1.6 (8)	C44—C39—P3—C33	−170.6 (4)
C30—C31—C32—C27	−1.2 (8)	C40—C39—P3—C33	7.9 (5)
C28—C27—C32—C31	1.1 (8)	C44—C39—P3—Cu1	−50.0 (4)
P3—C27—C32—C31	176.9 (4)	C40—C39—P3—Cu1	128.5 (4)
C38—C33—C34—C35	1.3 (7)	C38—C33—P3—C27	−17.6 (5)
P3—C33—C34—C35	173.5 (4)	C34—C33—P3—C27	170.6 (4)
C33—C34—C35—C36	−2.5 (7)	C38—C33—P3—C39	−125.8 (4)
C34—C35—C36—C37	2.2 (8)	C34—C33—P3—C39	62.4 (4)
C35—C36—C37—C38	−0.8 (8)	C38—C33—P3—Cu1	112.0 (4)
C36—C37—C38—C33	−0.3 (8)	C34—C33—P3—Cu1	−59.7 (4)
C34—C33—C38—C37	0.1 (7)	Cu1—O1—S1—O2	115.2 (3)
P3—C33—C38—C37	−171.7 (4)	Cu1—O1—S1—N1	−12.2 (3)
C44—C39—C40—C41	0.4 (7)	Cu1—O1—S1—C1	−127.9 (3)
P3—C39—C40—C41	−178.1 (4)	P1—N1—S1—O2	−157.4 (3)
C39—C40—C41—C42	−1.1 (8)	P1—N1—S1—O1	−27.6 (4)
C40—C41—C42—C43	1.0 (8)	P1—N1—S1—C1	88.2 (3)
C41—C42—C43—C44	−0.2 (8)	C6—C1—S1—O2	−18.7 (5)
C42—C43—C44—C39	−0.5 (8)	C2—C1—S1—O2	161.2 (4)
C40—C39—C44—C43	0.4 (8)	C6—C1—S1—O1	−141.2 (4)
P3—C39—C44—C43	179.0 (4)	C2—C1—S1—O1	38.7 (5)
Cu1—O3—P1—O5	100.4 (2)	C6—C1—S1—N1	98.7 (4)
Cu1—O3—P1—O4	−150.0 (2)	C2—C1—S1—N1	−81.5 (5)
